# Memory for melody and key in childhood

**DOI:** 10.1371/journal.pone.0187115

**Published:** 2017-10-27

**Authors:** E. Glenn Schellenberg, Jaimie Poon, Michael W. Weiss

**Affiliations:** 1 Department of Psychology, University of Toronto Mississauga, Mississauga, Ontario, Canada; 2 International Laboratory for Brain, Music, and Sound Research, Department of Psychology, Université de Montréal, Montréal, Québec, Canada; University of Zurich, SWITZERLAND

## Abstract

After only two exposures to previously unfamiliar melodies, adults remember the tunes for over a week and the key for over a day. Here, we examined the development of long-term memory for melody and key. Listeners in three age groups (7- to 8-year-olds, 9- to 11-year-olds, and adults) heard two presentations of each of 12 unfamiliar melodies. After a 10-min delay, they heard the same 12 *old* melodies intermixed with 12 *new* melodies. Half of the old melodies were transposed up or down by six semitones from initial exposure. Listeners rated how well they recognized the melodies from the exposure phase. Recognition was better for old than for new melodies, for adults compared to children, and for older compared to younger children. Recognition ratings were also higher for old melodies presented in the same key at test as exposure, and the detrimental effect of the transposition affected all age groups similarly. Although memory for melody improves with age and exposure to music, implicit memory for key appears to be adult-like by 7 years of age.

## Introduction

Melodies are abstractions, based on relations between consecutive notes in terms of pitch and time. Accordingly, changes in so-called “surface” features (i.e. key, timbre, and tempo) do not alter a melody’s identity even when they are readily perceptible. For example, adults easily identify a familiar song (e.g., *Yankee Doodle*) presented in a novel key, at a novel tempo, and in an unfamiliar timbre. The term relative pitch (RP) refers to the fact that memory for the pitches of melodies is based on relations (i.e., intervals) between consecutive tones, rather than actual pitch values. As such, the relation between tones with fundamental frequencies of 200 and 300 Hz is the same as that between tones of 300 and 450 Hz. In both instances, the higher tone has a frequency 1.5 times that of the lower tone (i.e., the difference in pitch between the first and second *Twinkles* of *Twinkle Twinkle Little* Star). RP allows individuals with no music training, who have no explicit knowledge of musical intervals, to recognize a familiar melody presented in a novel key, and to perceive when one note of a familiar song is sung sharp (too high) or flat (too low) in relation to tones that precede or follow it.

Absolute pitch (AP), by contrast, refers to the rare ability to remember the specific pitch of musical tones (e.g., middle C). Individuals with AP can produce and label individual tones presented in isolation, which means that they have memory for specific pitches *and* their names [1]. Nevertheless, even individuals without AP and no music training have memory for *key*, which refers to the pitch level of a piece of music rather than a single tone. For example, musically untrained adults are better than chance at identifying the original key of a familiar recording when contrasted with the same recording shifted by only one or two semitones [2]. When asked to sing a familiar pop song, most adults sing in a key that is close to the original pitch [3, 4]. In the present report, we use the term “key” interchangeably with pitch level. Accordingly, an octave transposition of a melody represents a 12-semitone change in pitch level (and key in this context), even though in *musical* terms the key would not change.

Adults also remember the key of novel melodies heard for the first time in the laboratory [5]. In one study [6], musically trained and untrained adults listened to a set of unfamiliar melodies, with each melody presented twice. After a delay of 10 min, 1 day, or 1 week, they heard the same (old) melodies as well as an equal number of novel melodies. Half of the old melodies were transposed upward or downward by six semitones (6 ST). Higher recognition ratings for old than for new melodies confirmed that listeners remembered the pitch relations that defined the tunes. In fact, there was no evidence of forgetting over the course of the week. Higher ratings for old melodies presented in the original key—compared to those that were transposed—confirmed that listeners also implicitly remembered the key. The negative effect of the transposition was evident after a 10-min and 1-day delay, but not after 1 week. In short, the results revealed two important findings: (1) adult listeners’ memory for melodies is relatively immune to changes in the delay between exposure and test, and (2) their mental representations include information about key for more than one but fewer than seven days.

In the present investigation, we asked whether memory for melody changes with age. Specific questions included: Do children form long-term memories for previously unfamiliar melodies rapidly, as adults do? Is memory for key evident in young children? If so, is memory for key stronger, weaker, or the same in childhood as it is in adulthood? These issues are important theoretically because scholars have proposed that infants begin life perceiving pitch absolutely [7, 8]. A change to RP processing is thought to occur during childhood, when familiar songs are heard in different keys, as, for example, when a father sings the same song much lower than a mother. One consequence is the putative rarity of AP, and the related notion that very few listeners form mental representations of music that contain absolute information about pitch.

In line with this perspective, 5-year-olds outperform adults in a task that requires them to identify a “special note” from a set of seven notes [9]. Infants also attend preferentially to the actual tones in a statistical learning task, whereas adults attend preferentially to relations between consecutive tones [7, 8]. The bias seen in infancy and childhood may be maintained if formal music training begins early in life. In fact, most individuals with AP started music lessons before the age of 7 [10]. Nevertheless, most individuals with early music training do *not* have AP, which contributes to its rarity and implicates a role for genetics or some other learning experience, such as speaking a tone language [1].

A different perspective holds that individuals perceive pitch absolutely and relatively across the lifespan. As with adults [2], 9- to 12-year-olds can identify the original key of familiar recordings [11]. Earlier in development, increases in age are predictive of *better* memory for key [12], although even infants remember the key of familiar melodies when natural stimuli are used (e.g., sung lullabies) [13], but not if the melodies are created electronically [14]. Other evidence documents that infants remember the pitch relations that define melodies, just as older individuals do. For example, in one study, 6-month-olds were exposed daily to a piano melody over the course of a week [14]. On the eighth day, they demonstrated a looking-time preference for a novel melody over the familiarized melody, even when the familiar melody was transposed.

Stalinski and Schellenberg [15] attempted to reconcile these different views by documenting that listeners of all ages perceive pitch absolutely *and* relatively, although a bias for absolute processing in early childhood becomes a bias for relative processing in adulthood. Five- to 12-year-olds and adults listened to pairs of melodies that were the same, different (reordered tones of the same melody), transposed, or different *and* transposed. Participants rated the similarity of each pair. Listeners of all ages rated transposed melodies and melodic changes to be less similar than identical melodies. For younger children, however, the transposition was more salient than the melodic change. With increases in age, the melodic change gradually became more salient than the transposition. By adulthood, a different melody was equally dissimilar whether it was transposed or not.

We know that infants and children remember the key of recordings they have heard multiple times in the same key [11–13], and that children remember novel melodies heard for the first time in the laboratory [16]. It is unclear, however, whether children remember the key of previously unfamiliar tunes. In the present study, we predicted that long-term melodic memory would improve with age, as in previous research that reported improvements in melody recognition from 5 to 10 years of age [16]. During middle childhood, implicit knowledge of conventions governing Western melodies improves with age [17], as does episodic memory in general [18], along with related improvements in working memory [19], mnemonic strategies [20], executive functions [21, 22], feature binding [23], and processing speed [24]. Dynamic Systems Theory holds that different aspects of behavior mature at different rates [25], such that multiple factors could lead to age-related differences in memory for key.

The children in the present study were 7 to 11 years of age. By age 7, children demonstrate an adult-like advantage for recognition of melodies presented in a vocal compared to an instrumental timbre [16]. Earlier in development, however, vocal melodies tend to be falsely “recognized” even when they are heard for the first time [16]. Thus, we did not test younger children, who focus on surface features at the expense of relations that define a melody. We tested a comparison group of adults, however, in order to document mature listeners’ performance with our stimulus melodies. We expected to replicate the finding that transposing the melodies from exposure to test would negatively impact recognition for adults [5, 6]. For children, the available literature allowed for three possibilities: compared to adults, the transposition could have a stronger detrimental effect, a weaker effect, or a similar effect.

## Materials and methods

The study was approved by the Research Ethics Board of the University of Toronto.

### Participants

The sample had 98 listeners recruited from a middle-class Canadian suburb. Participants were sampled from three age groups without regard to gender or music training. There were 32 7- to 8-year-olds (14 boys, 18 girls), 34 9- to 11-year-olds (14 boys, 20 girls), and 32 adults (5 men, 27 women). All participants had normal hearing, according to parent- and self-reports for children and adults, respectively. These age groups were chosen based on developmental changes in melodic processing, which have been documented with measures of perceptual similarity [15], recognition [16], and perceived emotion [26]. The group of younger children had a smaller age range than the older group because developmental change is particularly rapid earlier in life.

The 7- to 8-year-olds, hereafter *younger children*, had a mean age of 98 months (*SD* = 5); the 9- to 11-year-olds, hereafter *older children*, had a mean age of 132 months (*SD* = 11). The younger children had 1.0 years of music lessons on average (*SD* = 1.4); the older children had 1.7 years (*SD* = 1.8). In both instances, the distribution of music lessons was skewed positively. Music training was included as a between-subjects dummy variable in the statistical analyses because it can predict better long-term memory for novel melodies [6, 27, 28]. Specifically, younger children were considered to be musically trained if they had at least 1 year of music lessons (*n* = 13). Older children were classified as musically trained if they had at least 2 years (*n* = 15). An additional five children were recruited and tested but excluded from the final sample due to technical difficulties (*n* = 2) or experimental error (*n* = 3). Children received a gift card as a means of thanking families for participating.

Adults (age range: 16–20 years) were recruited from a freshman psychology course and received partial course credit. They had, on average, 5.9 years of music lessons (*SD* = 7.0). As with the older children, the adults were considered to be musically trained if they had at least 2 years of music lessons (*n* = 23). This classification scheme has been used previously when adults were recruited without regard to music training [5, 6, 29, 30].

More detailed information about music training for the three age groups is provided in S1 File.

### Stimuli

The 24 stimulus melodies were originally excerpted from collections of British and Irish folk tunes, recorded with MIDI in a piano timbre, and chosen because they were tonal (Western) sounding, relatively simple, yet unfamiliar [5, 6]. To accommodate the attention span of children, we reduced the duration of the test session by half (to 30 min) by shortening the melodies, which were approximately 30 s each (12–16 measures, three to four phrases), to 13–19 s, typically by eliminating the third and fourth phrases. Each melody was saved as a high-quality sound file twice: once with a median pitch of G4 (the G above middle C), and again with a median pitch of C#5 (6 semitones higher). Centering melodies in this manner ensured that different stimulus melodies at the low (or high) pitch level had notes drawn from different scales.

### Procedure

Participants were tested individually in a double-walled sound-attenuating booth. They sat in front of an iMac computer, which presented stimuli and recorded responses via customized software created with PsyScript [31]. The melodies were presented over headphones (Sony MDR-NC6). The procedure was similar to that used previously [5]. Before the test session began, participants heard multiple versions of *Happy Birthday* that differed in performance (surface) features (i.e., key and tempo), to demonstrate that a melody’s identity is invariant across such changes. Participants of all ages readily understood the point. The actual test session had an exposure phase, followed by a brief break and a recognition phase. In both phases, trials were self-paced by pressing the space bar, and ratings were made on 6-point numerical scales. The experimenter sat with children in the booth. Adult participants were tested independently.

In the exposure phase, participants heard 12 melodies, selected randomly from the set of 24. Each was presented twice, in one random order followed by a second random order (no direct repetitions). Half of the melodies (selected randomly) were presented in the lower key. The other half were presented in the higher key. For each presentation of each melody, participants were required to provide a liking rating to ensure that they listened to each melody.

In the recognition phase, participants heard all 24 melodies in random order. Their task was to rate whether they heard the tunes during the first part of the study. They were reminded that a tune’s identity is independent of changes in performance. The 12 new melodies were divided equally and randomly between the lower and higher keys. Six of the old melodies, selected randomly, were presented in the same key at test as exposure. The other six were transposed, with three of the previously higher and lower melodies (selected randomly) presented in the lower and higher keys, respectively. The design ensured that differences among melodies in inherent memorability did not influence the results, and that overall pitch height could not be used to identify whether a melody was old or transposed. For child participants, pictures were used instead of numbers to label the rating scale, with the smallest representing “definitely new” (corresponding to a rating of 1), and the largest representing “definitely old” (corresponding to a rating of 6). For adults, the rating scale had words and numbers. Between the exposure and test phases, there was a break of approximately 10 min during which children drew pictures with the experimenter, and adults completed a questionnaire that asked for demographic information and history of music training. A similar questionnaire was completed by a parent for each child.

## Results

For each listener, recognition ratings were converted to scores that measured area under the curve (AUC) [6]. AUC scores provide a bias-free measure of recognition accuracy. Recognition is considered perfect (AUC score = 1.0) when all ratings for old melodies are higher than all ratings for new melodies. For example, a listener has perfect recognition if all old melodies are rated 5 or 6 and all new melodies are rated 1 or 2 (no bias), or if all old melodies are rated 2 and all new melodies are rated 1 (a conservative bias). In both instances, ratings perfectly distinguish old from new melodies. A score of 0.5 indicates chance performance, such that ratings for old and new melodies are indistinguishable (no recognition).

**Fig 1:**
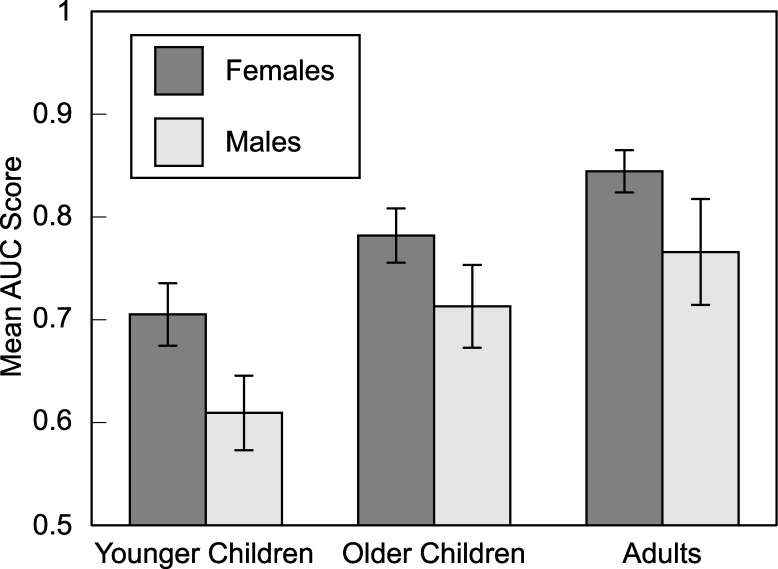
Mean overall AUC scores as a function of age group and gender. Adults recognized the melodies better than older children, who had better recognition than younger children. Across age groups, female participants had better recognition than males. Error bars are standard errors.

An overall AUC score was calculated for each participant. Descriptive statistics are illustrated in Fig 1. Data are provided in S2 File. One-sample *t*-tests confirmed that performance was significantly better than chance for adults, *t*(31) = 17.01, *p* < .001, older children, *t*(33) = 10.95, *p* < .001, and younger children, *t*(31) = 6.66, *p* < .001. A three-way ANOVA, with age group, gender, and music training as independent variables, confirmed that there was a difference between groups, *F*(2, 86) = 7.08, *p* = .001, η^2^ = .112. Adults performed better than older children, *p* = .036, and younger children, *p* < .001, and older children performed better than younger children, *p* = .013 (Tukey’s test). Females also outperformed males, *F*(1, 86) = 8.31, *p* = .005, η^2^ = .065. There was no main effect of music training, *p* > .2, no two-way interactions, *F*s < 1, and no three-way interaction, *p* > .2.

**Fig 2:**
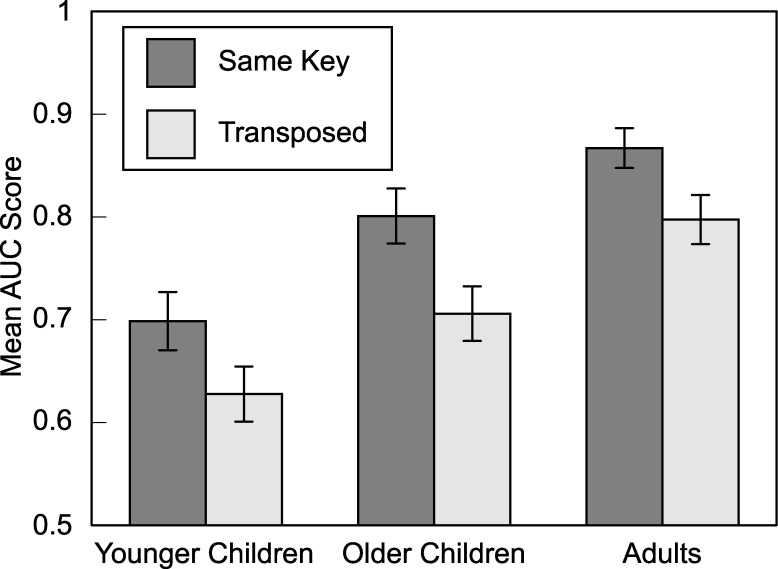
Mean AUC scores for same-key and transposed melodies as a function of age group. Although melody recognition improved with age, the detrimental effect of the transposition was similar across age groups. Error bars are standard errors.

We then asked whether some old melodies were remembered better than others, specifically if those presented in the same key at test and exposure were recognized better than those that were transposed. For each listener, we calculated AUC scores separately for old-same and old-transposed melodies. Descriptive statistics are illustrated in Fig 2. A mixed-design ANOVA with key (same or transposed) as a repeated measure and age group, gender, and music training as between-subjects variables uncovered a main effect of key, *F*(1, 86) = 23.94, *p* < .001, partial η^2^ = .218. Old melodies that were reheard in their original key (*M* = .79, *SD* = .16) were remembered better than their transposed counterparts (*M* = .71, *SD* = .16). Main effects of age, *p* = .001, and gender, *p* = .005, were again evident, but there was no hint of a two-way interaction between key-change and age, and no other two-way interactions, *F*s < 1. In other words, transposing melodies from exposure to test had a similar detrimental effect on recognition for all listeners. There were also no higher-order interactions, *p*s > .1.

Finally, correlational analyses revealed that the magnitude of the detrimental effect of the key change (AUC same—ACU transposed) was unrelated to memory for melodies (AUC overall scores) for the entire sample, *p* > .7, and when each of the three age groups was examined separately, *p*s > .1.

## Discussion

We examined long-term memory for melody and key in children and adults. As expected, adults recognized previously unfamiliar melodies better than 9- to 11-year-olds, who had better recognition than 7- to 8-year-olds. This developmental progression is likely to stem from increased informal exposure to music, and from cognitive development more generally. Although it is impossible to tease apart effects of informal exposure to music from those of maturity, by 4 years of age, musically untrained children demonstrate Western-specific knowledge of key and harmony [32], which must be learned. Similarly, Western 6-month-olds are equally adept at processing and remembering meters that are common or uncommon in their musical environment [33], but by 12 months of age, performance is better with the common meters [34]. In short, passive exposure leads to enhanced processing of music that is relevant in a listener’s native environment. Better encoding and retrieval of our stimulus melodies would also stem from age-related improvements in general cognitive abilities, including long-term, working, and short-term memory [35], executive functions [21, 22], feature binding [23], and processing speed [24].

In prior research, adult listeners recognized previously unfamiliar melodies equally well whether the delay between exposure and test was 10 min, 1 day, or 1 week, and this result was evident in three different samples, each with approximately 100 participants [6]. Future research could determine if younger listeners show the same continuity over time. If so, developmental differences in memory for melodies would be similar regardless of the delay. Alternatively, age-related advantages in recognition could become larger or smaller as the delay increases.

The other major finding was that implicit memory for key was evident in all age groups. Old melodies that were in the same key at exposure *and* test were recognized better than melodies that were transposed. Moreover, the transposition negatively affected recognition similarly regardless of age, gender, or music training. This finding is consistent with results from previous studies that examined memory for the key of familiar recordings heard multiple times over a relatively long time period. Although methods and stimuli varied across studies, performance in absolute terms was similar for children and adults [2, 11, 12].

Implicit memory for key was unrelated to explicit memory for melody, which implicates separate developmental processes. In general, implicit musical knowledge appears early in development and is relatively impervious to individual differences in age and music training. For example, when Western listeners hear a sequence of chords in an established musical key, they expect the final chord to be the tonic (the chord based on *do*). Accordingly, when asked to judge whether the final chord is performed with a specific timbre, sung with a particular vowel, or consonant or dissonant, performance is slower or less accurate when the chord is the sub-dominant (based on *fa*) instead of the tonic [36]. Such *harmonic priming* effects are evident in musically trained and untrained children [37] and adults [38], and even in a patient with a severe deficit in music perception (i.e., *amusia*) [39]. Although children find key changes more salient than do their adult counterparts [15], such age differences may be limited to tasks that rely on short-term memory, when the transposition in a same-different task is particularly noticeable, and to other measures of explicit musical knowledge.

Why would young listeners have implicit memory for key? On the one hand, memory for key seems counter-productive (i.e., a failure to generalize) because the identity of a melody is independent of pitch level. Moreover, nonhuman species tend to perceive and remember specific pitches rather than pitch relations [40]. On the other hand, by middle childhood, familiar recordings (e.g., themes from TV shows) have been heard multiple times at exactly the same pitch level. Singing to infants is also a universal behavior [41], and there is more pitch consistency in infant-directed singing than in infant-directed speech, with songs varying by less than one semitone on average from one week to the next [42]. Thus, memory for pitch level (or key) would enhance memory for auditory signals in general and for a caregiver’s voice in particular. Additional evidence suggests that encoding invariant features of vocalizations offered an evolutionary advantage to our ancestors. For example, identifying a speaker’s voice involves encoding pitch and formant information, which contribute to evaluations of the speaker’s attractiveness [43]. Averaging utterances across voices yields a more attractive utterance, in part due to clearer pitches brought about from reduction of aperiodic noise, but also because shifting the pitch of a voice toward the average pitch of its gender increases attractiveness [44]. An unusually high pitch can also be used to identify when another person is angry, scared, or happy, whereas a low pitch indicates sadness or tenderness [45]; effective use of these cues requires memory for the speaker’s baseline pitch. In short, music listening may co-opt adaptive mechanisms associated with processing vocalizations, mate selection, and identifying speakers and their intentions.

We found no effect of music training on memory for melody or key. In previous research, music training had marginal or inconsistent associations with long-term memory for melodies but not for key [5, 6]. In other studies that examined long-term memory for melody with no transpositions from exposure to test [46], similarly inconsistent results emerged, with better recognition for musically trained than untrained participants in some instances [6, 27, 28] but not in others [47–49]. In tests of short-term memory for melodies presented in transposition, however, music training predicts good performance more reliably [29, 50]. Thus, Halpern and Bartlett’s [46] review of melodic memory concluded that recognition advantages based on music training are most likely to be evident in short-term memory tasks that require the listener to make relatively fine but musically relevant distinctions. It is nevertheless possible that effects of music training could emerge on the present task if highly trained musicians were recruited. These individuals tend to have enhanced RP perception, which allows them to detect very small mistunings to melodies [51]. It is therefore possible that professional musicians might show less of a decrement (or no decrement) in recognition when a melody is transposed from exposure to test.

Gender was associated with memory for melody but not with memory for key, with females exhibiting better melody recognition than males across age groups (Fig 1). In previous research, female advantages emerged in some music tasks, such as those that require young children to identify emotions conveyed musically [26]. These associations are not always reliable [52], however, and can disappear among older children and adults [26]. In any event, gender was of no theoretical interest. Moreover, gender had no bearing on implicit memory for key, which appears to be remarkably consistent across individuals.

The present study is not without limitations. For example, the stimulus melodies were drawn from a single musical genre, such that it is unclear whether the results would generalize to stimuli drawn from other Western genres or from non-Western music. The sample was similarly restricted, to individuals from middle-class families from a Canadian suburb. Finally, as noted above, the delay between exposure and test was short (approximately 10 min) so that participants needed to visit the laboratory only once. It is therefore unknown whether children’s memory for key would be evident after longer delays, as it is for adults [6].

In sum, the present findings converge with others indicating that the unusual ontogeny of AP stems from the rare ability to attach arbitrary labels to isolated tones, and not from a lack of memory for the pitch level of ecologically valid music. After hearing previously unfamiliar melodies only twice in the laboratory, even 7-year-olds remembered the stimulus tunes explicitly and their key (or pitch level) implicitly. Our failure to uncover age differences in implicit memory for key did not appear to be a consequence of a lack of power because robust age-related improvements were evident for explicit memory for melody. The results add to our knowledge of human musicality and how it develops as a function of aging and music listening. They also confirm that much knowledge of music is acquired implicitly, such that appropriate tasks are necessary to determine the extent and limits of implicit musical knowledge [36]. Future research could use similar methods to vary the magnitude of the transposition, the delay between the exposure and recognition phases, or surface features other than key (e.g., tempo, timbre).

## Supporting information

S1 File Details about music training. (DOCX)

S2 File Dataset. (CSV)
