# Supplementary material for: Memory for melody and key in childhood
Source: PLoS One. 2017 Oct 27;12(10):e0187115. doi: 10.1371/journal.pone.0187115 (PMC5659795; doi:10.1371/journal.pone.0187115)
Supplement: S1 File — (DOCX) [file pone.0187115.s001.docx]

**Supplementary Materials**

**Additional Information About Music Training and Demographics**

1. ***Were participants still practicing their instruments?***

Among musically trained adults, 4 participants out of 23 were still practicing their instruments. Ten of 15 older children who were musically trained were taking lessons at the time of testing, as were 9 of 13 younger children.

1. ***What types of music education pedagogies were used in those that qualified as musicians? How were individuals trained?***

We make no claims that any participant was a musician according to objective criteria.

Across all age groups, music lessons were taught in individual or group settings.

Adults with at least 2 years of formal and informal training were categorized as musically trained. Formal training included private one-on-one lessons or group lessons at local music schools and centers. Pedagogies included instruction based on the Royal Conservatory of Music (RCM) syllabi or middle-school/high-school curricula (e.g. band class). Participants with 2 years or more of informal lessons (e.g., self-taught) were also considered to be musically trained.

Children who were classified as musically trained were instructed in individual or group settings under a variety of pedagogies (e.g., RCM, Suzuki, Yamaha).

1. ***Did participants learn to read music? Were the children involved fluent music readers or where they trained by rote?***

We did not collect data about the ability to read music notation.

Theory training for musically trained adults took place in individual or group settings. Most learned theory in middle- and high-school music classes (n=7), and could presumably read music. Participants who did not indicate a background in theory were assumed to have achieved basic note reading and notation skills in their instrumental training, particularly those trained using the RCM syllabi.

Although background in music theory was not indicated in the child questionnaire, musically trained children were assumed to have acquired a basic foundation in note reading during their instrumental training.

1. ***What instruments did participants play and for how long?***

For each participant, duration of music training was calculated as the sum of instruction for all instruments (including voice) that they had studied.

Overall, musically trained adults had vocal lessons and lessons on brass, percussion, strings, and woodwind instruments, for durations that ranged from 1 month to 15 years.

Musically trained older children were trained primarily in piano (*n*=11), with fewer children studying voice (*n*=3) and guitar (*n*=2). Other children were trained in woodwind, percussion, and stringed instruments (e.g., oboe, drums, and violin). Experience on an instrument ranged from 6 to 61 months.

The majority of 7 younger children also received training on piano (*n*=12). Others studied voice (*n*=3), guitar (*n*=1), and violin (*n*=1). Duration of training ranged from 5 to 60 months.

- Adults

Instrument: Range of experience in years (trained participants only)

- - - Alto Sax: 4
    - Bass Clarinet 1
    - Clarinet: 1-2
    - Drums: 1-2
    - Euphonium: 3
    - Flute: 1-9
    - French Horn: 8
    - Guitar: 1-5
    - Oboe: 1
    - Piano: 0.08-14
    - Recorder: 1-4
    - Saxophone: 1-5
    - Trombone: 1-4
    - Trumpet: 2-3
    - Tuba: 1
    - Violin: 4
    - Voice: 2-15
- Children
  - Older children

Instrument: Range of experience in months (trained participants only)

- - - Piano: 12-60
    - Drums: 4
    - Flute: 28
    - Guitar: 6-72
    - Oboe: 14
    - Trombone: 14
    - Various: 20
    - Violin: 12
    - Voice: 12-31
  - Younger children

Instrument: Range of experience in months (trained participants only)

- - - Piano: 8-60
    - Guitar: 12
    - Violin: 50
    - Voice: 5-12

1. ***What was the socioeconomic status of the population?***

Child participants predominantly came from middle to upper-middle class families. Family incomes ranged from 25,000 to more than 200,000 earnings per year. Most families had annual incomes of $125,000-150,000, with most parents holding an undergraduate degree. In short, all child participants came from relatively enriched environments and were involved in a diversity of extracurricular activities both musically related (e.g. dance and drama) and unrelated (e.g. sports).

Adult participants were not required to indicate their socioeconomic background. Nevertheless, in Canada, individuals tend to attend universities that are located close to their family homes, in contrast to the United States, where there is a tradition of “going away to college.” Thus, we presume that most of the adult participants also came from middle to upper-middle class backgrounds.
